# Moderate Physical Activity Mediates the Association between White Matter Lesion Volume and Memory Recall in Breast Cancer Survivors

**DOI:** 10.1371/journal.pone.0149552

**Published:** 2016-02-25

**Authors:** Gillian E. Cooke, Nathan C. Wetter, Sarah E. Banducci, Michael J. Mackenzie, Krystle E. Zuniga, Elizabeth A. Awick, Sarah A. Roberts, Brad P. Sutton, Edward McAuley, Arthur F. Kramer

**Affiliations:** 1 Beckman Institute for Advanced Science and Technology, University of Illinois at Urbana-Champaign, Urbana, IL, United States of America; 2 Department of Behavioral Health and Nutrition, College of Health Sciences, University of Delaware, Newark, DE, United States of America; 3 School of Family and Consumer Sciences, Texas State University, San Marcos, TX, United States of America; 4 Department of Kinesiology & Community Health, University of Illinois at Urbana-Champaign, Urbana, IL, United States of America; 5 Department of Psychology, University of Illinois at Urbana-Champaign, Urbana, IL, United States of America; 6 Department of Bioengineering, University of Illinois at Urbana-Champaign, Urbana, IL, United States of America; University Of São Paulo, BRAZIL

## Abstract

Increased survival rates among breast cancer patients have drawn significant attention to consequences of both the presence of cancer, and the subsequent treatment-related impact on the brain. The incidence of breast cancer and the effects of treatment often result in alterations in the microstructure of white matter and impaired cognitive functioning. However, physical activity is proving to be a successful modifiable lifestyle factor in many studies that could prove beneficial to breast cancer survivors. This study investigates the link between white matter lesion volume, moderate physical activity, and cognition in breast cancer survivors following treatment compared to non-cancer age-matched controls. Results revealed that brain structure significantly predicted cognitive function via mediation of physical activity in breast cancer survivors. Overall, the study provided preliminary evidence suggesting moderate physical activity may help reduce the treatment related risks associated with breast cancer, including changes to WM integrity and cognitive impairment.

## Introduction

Breast cancer is the leading cancer type in women; in 2015, 29% of new cancer cases in the United States are estimated to be breast cancer related [1]. The risk for developing breast cancer increases with age [2], with 1 in 8 women developing invasive breast cancer. While the median age for diagnosis is 61, long-term effects of breast cancer treatment are emerging [3]. Although breast cancer is among the principal causes of cancer-related deaths [1,4,5], increased knowledge of breast cancer in the general population, earlier detection, and improvements in treatments offered has greatly impacted survival rates. The consequence is that breast cancer survivor’s (BCS) often have to deal with cognitive impairments long after the cessation of treatment, at great cost to both themselves and society. The disease and treatment greatly impact many facets of the BCS life, in particular, there are well-documented reports of impairments in cognitive function both at diagnosis, and after treatment [6–8]. This may reflect a pattern of accelerated aging in the brain, with reported difficulties in executive function, processing speed, memory and attention [9,10]. Accordingly, researchers are beginning to investigate whether treatment for breast cancer augments age-related changes in the function and structure of the brain [11]. Recent momentum to examine the structural changes in the brain accompanying these impairments has revealed alterations in both gray and white matter, proposing a possible link between structural changes in the brain and functional deficits in cognition [12–15]. There are several factors linked to the deficits associated with breast cancer treatment, including time since diagnosis, age at onset of disease, and lifestyle factors.

Although some studies find no differences following breast cancer treatment [16], many report significant changes in the structure and function of the brain in BCS for years or even decades following treatment [10,17–20]. Comparing patients treated with chemotherapy and surgery (C+) versus surgery only (C-) and non-cancer controls (NCC), Deprez and colleagues reported significant cognitive and structural changes associated with chemotherapy [10,21]. Patients were assessed after surgery but before chemotherapy–and then tested 3–5 months after cessation of chemotherapy. Within the C+ group, cognitive performance declined significantly with treatment, but no such differences were evident in the C- group. There was also a significant interaction between group and performance in memory and processing speed tasks, with the C+ group performing significantly worse than the C- and NCC groups. Additionally, within the C+ group there was a significant relationship between change in memory performance and change in white matter (WM) integrity; greater declines in WM integrity were related to greater cognitive decline. Interestingly, there were no significant differences in either patient group at baseline (versus NCC), suggesting no impact of cancer diagnosis alone. These findings suggest that alterations in the microstructure of WM are evident following chemotherapy treatment for breast cancer. Importantly, these alterations in WM integrity may impact the transmission of information between regions of grey matter that support cognitive function [22]. Deprez and colleagues suggest that damage to these WM regions involved in transfer of information around the brain, reduces the efficiency of interaction among different neural systems [23]. While they propose that the treatment related cognitive impairments in cancer are related to alterations in the myelin structure of WM, they indicate that further investigation is needed to confirm this hypothesis.

De Ruiter and colleagues reported cognitive impairments, reduced WM integrity, increased axonal injury and reduced gray matter (GM) volume in a small group of C+ patients when compared to C- group up to 10 years after treatment [24]. They suggest this reflects long-term damaging effects that chemotherapy has on the white and gray matter of the brain. This group also extended the length of time since treatment cessation to over 20 years in 180 C+ women; both total brain volume and GM volume were significantly lower in the C+ group [17,25]. While they did not find differences in WM integrity between NCC and C+, within the C+ group they did find a negative relationship between time since cessation of chemotherapy and WM integrity–suggesting that WM integrity in chemotherapy treated BCS deteriorates with time since treatment. Long-term follow-ups are necessary to determine if WM integrity recovers with time.

There are well-documented changes in brain structure and function with aging [26–34]. Given that recent research has suggested that treatment for breast cancer may resemble an accelerated pattern of aging in the brain [35,36], the impact of age at diagnosis could differ across the lifespan [9,37,38]. Mandelblatt and colleagues examined the shared underlying pathways of cancer-related cognitive impairment and aging, including changes in hormone levels, inflammation and decreased blood flow [9]. In particular, they discuss the impact of low physical activity (PA) and decreased cognitive function. The link between PA and adult hippocampal neurogenesis has received a lot of attention; increased PA seems to stimulate precursor cells, the cells from which adult neurogenesis derives [39]. The ‘neurogenic reserve hypothesis’ proposed by Kempermann and colleagues suggest that an absence of PA early in life can only be somewhat counteracted later. They believe that it is imperative that we maintain a continually active lifestyle, sustaining the capacity for adult neurogenesis. Furthermore, they discuss a two-pronged issue for people with low levels of PA–not only do they have less plasticity, but their ability to achieve long-lasting benefits from this plasticity is diminished [39]. Consequently, PA is proving to be a successful modifiable lifestyle factor across the lifespan in both healthy and clinical populations [32,33,40–46]. A large aging study that predicted changes in brain structure based on earlier PA (3 years prior), reported that higher levels of self-reported PA predicted less atrophy in both GM and WM, and lower WM lesion volume [47]. Extending this work to include objective measures of PA, Burzynska and colleagues showed that lower WM lesion volume in older adults was related to greater moderate-vigorous PA [44].

While breast cancer may result in accelerated aging of the brain, and aging brains benefit greatly from increased PA, considerably less is understood about the link between PA, cognition and breast cancer. PA levels often change after a diagnosis of breast cancer; with up to an 11% decrease in PA within a year of diagnosis, though these decreases are considerably higher in women treated with radiation and chemotherapy [48]. The danger is that BCS may not return to their pre-diagnosis levels of PA, increasing their risk for weight gain and associated cardiovascular problems. BCS often report exercise intolerance following treatment for breast cancer [49], with many spending only 2% of their awake time in moderate-vigorous PA, and 80% of their time sedentary [50]. A novel study highlighting the importance of PA reported that 30 days of bed rest resulted in a greater reductions in fitness then 30 years of aging [51,52]. In response to the growing need to encourage continued and even increased PA among cancer survivors, the American College of Sports Medicine published guidelines [53] that recommend BCS progress up to 150 minutes of moderate PA per week. While several studies have highlighted the benefits of physical activity in BCS [40,54,55], only two studies have explicitly examined the association between PA/fitness and cognition in BCS [55,56]. Both studies reported a significant relationship between physical activity and cardiorespiratory fitness (CRF) and cognition in BCS. Although there is a lack of RCTs examining the effects of PA on cognitive function in BCS, these studies do highlight some of the potential benefits.

We were interested in examining the role that PA plays in the structure and function of the brain following treatment for breast cancer. Our primary objectives were: (a) to determine whether BCS and non-cancer age-matched controls differed in memory recall, WM lesion volume and PA and (b) to examine the relations among these measures and the extent to which PA mediated the relations between WM lesion volume and memory recall.

## Methods

### Participants

BCS were primarily recruited through an oncology clinic, though some were recruited, along with the non-cancer age-matched controls, through local advertisements. After expressing initial interest, women were contacted by phone and provided a full study description. During the initial contact, interested individuals completed a demographics questionnaire and a personal medical history questionnaire, including self-reported information on breast cancer diagnosis and treatment history (BCS only). Of the 141 total contacts, 73 consented, and 11 women withdrew after consenting owing to schedule conflicts (n = 2), no longer interested (n = 3), or unable to contact (n = 6). Furthermore, some women only underwent surgery (n = 3) and were excluded from these analyses, and an additional participant was identified as an outlier (see below). This resulted in a final sample of 58. Participants were female BCS who had undergone surgery for breast cancer and had completed primary treatment (chemotherapy, radiation therapy or both) within the past 3 years (n = 30), or age-matched controls with no prior diagnosis of cancer (n = 28).

Given the likely interaction between time since cessation of therapy and age at onset of disease, we recruited BCS within 3 years of completion of treatment and aged between 18–70 years. Inclusion criteria for all included English speaking, normal/corrected to normal vision, no current use of brain training games (e.g. Lumosity ®, BrainHQ ®), no history of stroke, transient ischemic attack, or surgery that involved removal of brain tissue, not currently pregnant, a score of ≥ 26 on the modified Mini-Mental Status Exam– 2^nd^ Edition (MMSE-2), and able to walk on a treadmill unaided. All participants provided written informed consent and were required to provide written consent from a primary care physician or oncologist indicating they were cleared to participate in the cardiorespiratory fitness, magnetic resonance imaging (MRI) and cognitive testing sessions. Participants completed an initial visit for cardiorespiratory fitness assessment and 7 days of accelerometer monitoring. During a second visit participants completed cognitive testing followed by a third visit to complete a series of MRI scans. The study was approved by the University of Illinois Urbana Champaign Institutional Review Board.

### Physical Activity

Physical activity (PA) was determined using 7-day accelerometer monitoring (model GT3X, Actigraph: Pensacola, FL). Participants were asked to wear the accelerometer during waking hours and wear time was recorded on an accelerometer log. Data were downloaded and digitally converted to “activity counts” per minute (i.e., one epoch), and processed using MeterPlus 4.2 software (Santech Health: San Diego, CA). Only days with at least 10 valid hours of wear time were included in the analysis, and hours with greater than 60 min of consecutive zeros were considered invalid (i.e., non-wearing). Activity counts were summed and averaged across the total number of valid days for a total daily activity score. Average daily moderate PA was calculated by summing the total valid moderate activity counts, then dividing by the number of valid days.

### Body Mass Index (BMI)

Height and weight were measured using a Seca electronic scale and stadiometer (Model 763 1321139, Chino, CA). Participants were measured while wearing light clothing and without shoes. BMI was calculated using the standard formula of weight (kg) / height (m)^2^.

### Mini-Mental Status Exam – 2^nd^ Edition (MMSE-2)

The standard version of the MMSE-2 which allows for a brief standardized assessment of cognitive status was used to screen for signs of cognitive impairment [57]. It examines orientation, attention, memory, confrontation naming, language, comprehension and motor function. Participants scoring < 26 out of a possible 30 were excluded.

### Story Memory

The expanded version of the MMSE-2 includes an immediate story recall–participants were read a short story aloud and asked to recall the story using the same words. There are 25 elements to the story, and a credit was given for each one successfully recalled. The story memory was designed to evaluate verbal explicit learning and verbal free recall [57].

### MRI Protocol

All images were acquired during a single session on a 3 T Siemens Trio Tim system (Siemens, Erlangen, Germany). High-resolution structural MR scans were acquired using a 3D magnetization prepared rapid acquisition gradient echo (MPRAGE) T1-weighted sequence (TR = 1900 ms; TE = 2.32 ms; TI: 900 ms; flip angle = 9°; matrix = 256 × 256; FOV = 230mm; 192 slices; resolution = 0.9 × 0.9 × 0.9 mm; GRAPPA acceleration factor 2). While the lesion volumes were acquired using a 3D, variable flip angle turbo spin echo sequence with fluid-attenuated inversion recovery (FLAIR) (TE 388 ms, TR 6000 ms, TI 2200 ms, FOV 250x250x160 mm, 1 mm isotropic sampling; GRAPPA acceleration factor 2).

### White Matter Lesion Protocol

We used previously developed open-source software based on the image processing toolkit FSL (functional magnetic resonance imaging of the brain (FMRIB) Software library) to perform automated computation of lesion masks and volumes [58,59]. We accomplished this by using BET (Brain Extraction Tool) [60] to extract the brain, then used FAST (FMRIB's Automated Segmentation Tool) [61] on the result, with two tissue classes, brain and non-brain. Hyper intense regions were classified as non-brain tissue and we examined the histogram of the non-brain tissue to identify the lesions. We then used standard space masking with nonlinear registration via FNIRT (FMRIB's Nonlinear Image Registration Tool) [62] to eliminate false positives that consistently appear in certain parts of the brain, such as the septum pellucidum. The final output was total lesion volume, expressed as a percentage of total brain volume [59].

### Data Analysis

All analyses were performed using IBM SPSS version 22 (IBM, 2013), and data is provided in S1 Dataset. Frequency distributions were examined to check for missing information and out-of-range values. Tests of normality were run on the whole brain WM data (lesion volume corrected for percent of whole brain volume), story memory recall and moderate PA data–as a result of these tests, lesion volume was transformed using log transform. We excluded subjects with extreme values on any of these measures (3 SD beyond mean). One subject had extreme moderate PA activity in the non-cancer age-matched control group and was dropped from all analyses.

Given the small sample size, analyses were conducted across all BCS in comparison to non-cancer age-matched controls. To investigate these differences multivariate ANCOVAs were conducted on background demographics, lesion volume, moderate PA and story memory recall. Effects sizes, partial Eta squared, were calculated and reported. We explored associations between lesion volume, PA and story memory using partial correlations, controlling for age and years of education. Further analyses in the BCS group also included time since cessation of treatment.

**Fig 1:**
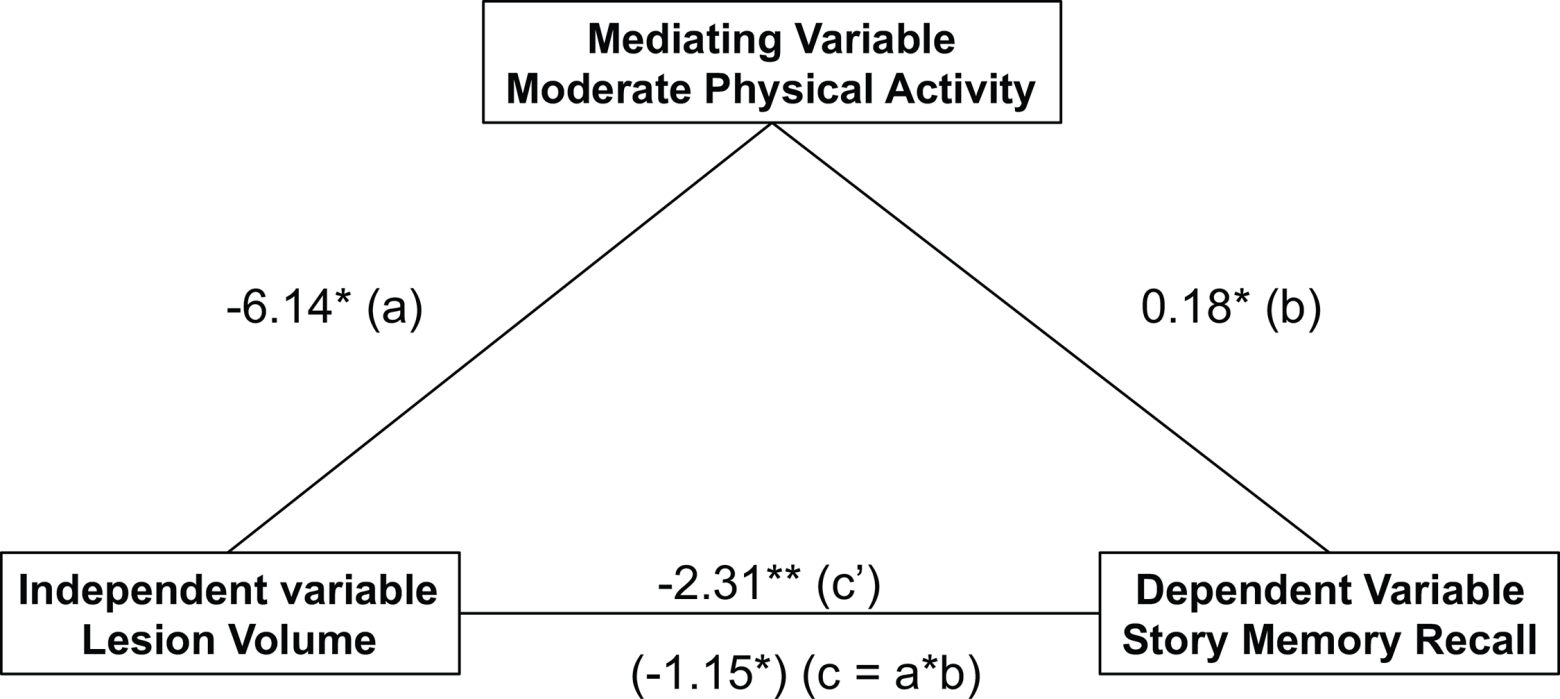
Mediation Diagram with unstandardized coefficients.

Where significant relationships were found, a simple mediation analysis was conducted using ordinary least squares path analysis [63], controlling for covariates (PROCESS toolbox in SPSS). This analysis examined whether moderate PA mediated the relationship between lesion volume and story memory recall (see Fig 1). While there is value in examining the relationship between an independent and dependent variable, this relationship can be overemphasized before controlling for a mediator, leading to misinformed results [64].

Moderate physical activity significantly mediates the relationship between lesion volume and story memory recall in breast cancer survivors.

## Results

### Participant Characteristics

There were no significant differences between the groups (n = 58) on background characteristics such as age, years of education and BMI, nor were there significant differences on lesion volume or moderate PA; however, there were differences on levels of story recall (f = 8.929, p = 0.004, df = 1, 56), with BCS remembering significantly fewer details (Table 1). Specifics about disease and treatment characteristics of the BCS are presented in Table 2. Older participants had greater lesion volume (r = 0.563, p = 0.001), while those who were more educated were also likely to engage in greater levels of moderate PA (r = 0.432, p = 0.001), accordingly, both variables were included as covariates throughout all analyses.

**Table 1:** Participant Characteristics.

Variable	Breast Cancer Survivors (n = 30)	Non-Cancer Controls (n = 28)	Sig	Partial Eta Squared
**Age (years)**	**55.83 (1.74)**	**55.43 (1.80)**	**0.872**	**0**
**Education (years)**	**16.40 (0.53)**	**16.63 (0.55)**	**0.769**	**0.002**
**MMSE**	**29.27 (0.20)**	**29.18 (0.21)**	**0.762**	**0.002**
**Lesion Volume (mm3)**	**1171.05 (264.35)**	**1070.38 (273.62)**	**0.792**	**0.001**
**Story Memory Recall**	**12.97 (0.82)**	**16.50 (0.85)**	0.004**	**0.138**
**Body Mass Index**	**27.47 (1.01)**	**28.32 (1.04)**	**0.564**	**0.006**
**Moderate PA**	**15.81 (2.67)**	**20.61 (2.76)**	**0.217**	**0.027**

Participant mean (SE) demographic, Mini-Mental Status Exam (MMSE), average daily moderate physical activity (PA), lesion volume, and memory data by group (breast cancer survivors, healthy controls) (including p-values of multivariate ANOVAs and effect size using partial Eta squared). Significant p-values were reported at p < 0.01**.

**Table 2:** Disease and treatment characteristics of breast cancer survivors (n = 30).

**Stage**	DCIS	5 (16.7)
	Stage I	8 (26.7)
	Stage II	11 (36.7)
	Stage III	3 (10)
** **	Unknown	3 (10)
**Estrogen Receptor Positive N (%)**	Yes	23 (76.7)
	No	6 (20)
** **	Unknown	1 (3.3)
**Adjuvant Treatment N (%)**	Surgery	30 (100)
	Radiotherapy Only	11 (36.7)
	Chemotherapy Only	8 (26.7)
** **	Chemotherapy Plus Radiation	11 (36.7)
**Months Since End of Treatment**	Radiotherapy Only	13.14 (2–32)
**Mean (Range)**	Chemotherapy Only	20.38 (7–33)
** **	Chemotherapy Plus Radiation	15.86 (3.5–31)

DCIS, ductal carcinoma in situ.

### Relationship between lesion volume, physical activity and memory recall

**Fig 2:**
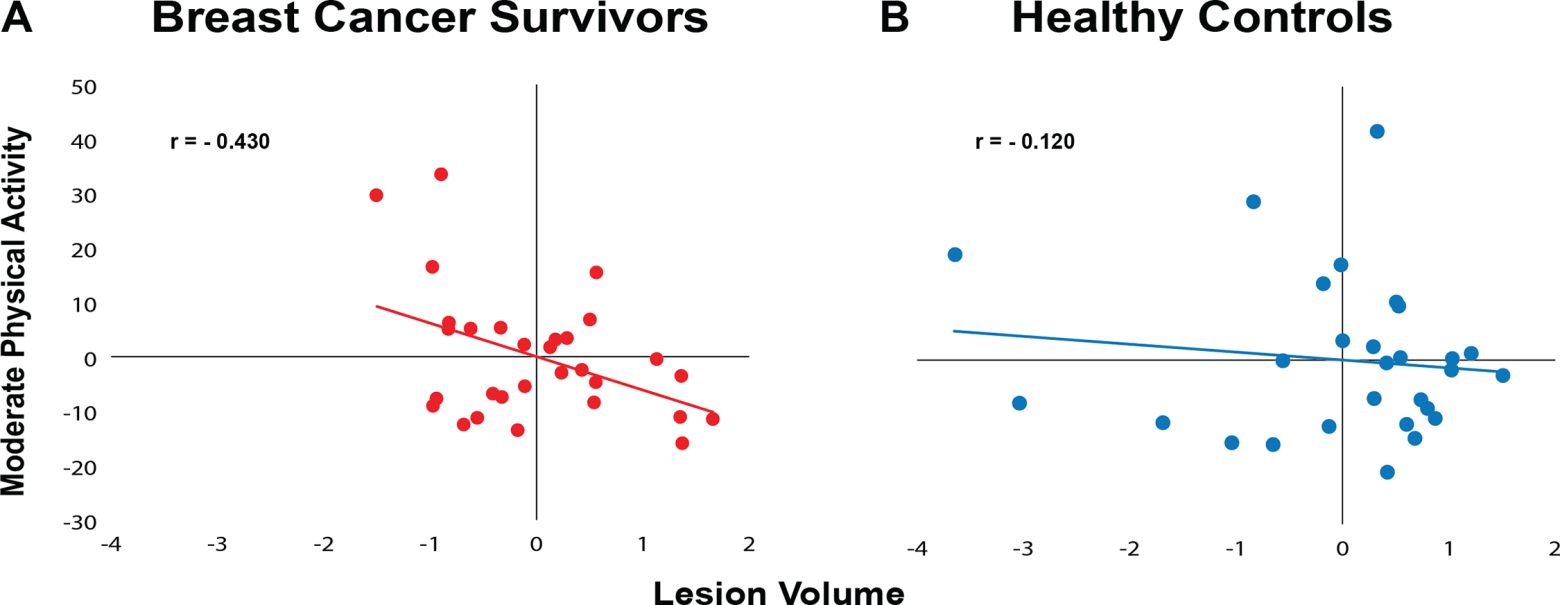
Scatterplots of unstandardized coefficients examining the relationship between moderate physical activity and lesion volume. Controlling for age and education, there was a negative relationship between moderate physical activity and lesion volume in breast cancer survivors; greater moderate physical activity was significantly related to lower lesion volume (p<0.05), while there was no relationship in the non-cancer age-matched controls (p = 0.280).

**Fig 3:**
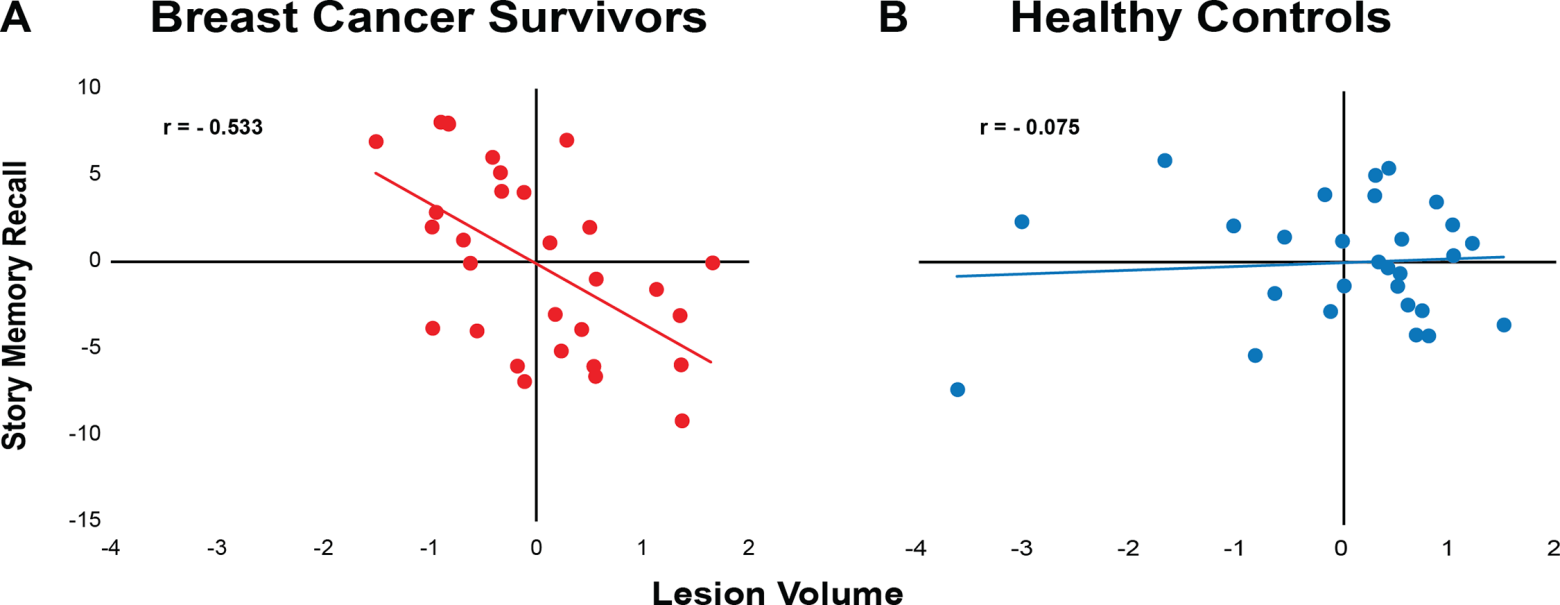
Scatterplots of unstandardized coefficients examining the relationship between story memory recall and lesion volume. Controlling for age and education, there was a negative relationship between story memory recall and lesion volume in breast cancer survivors; greater story memory recall was significantly related to lower lesion volume (p<0.01), while there was no relationship in the non-cancer age-matched controls (p = 0.358).

**Fig 4:**
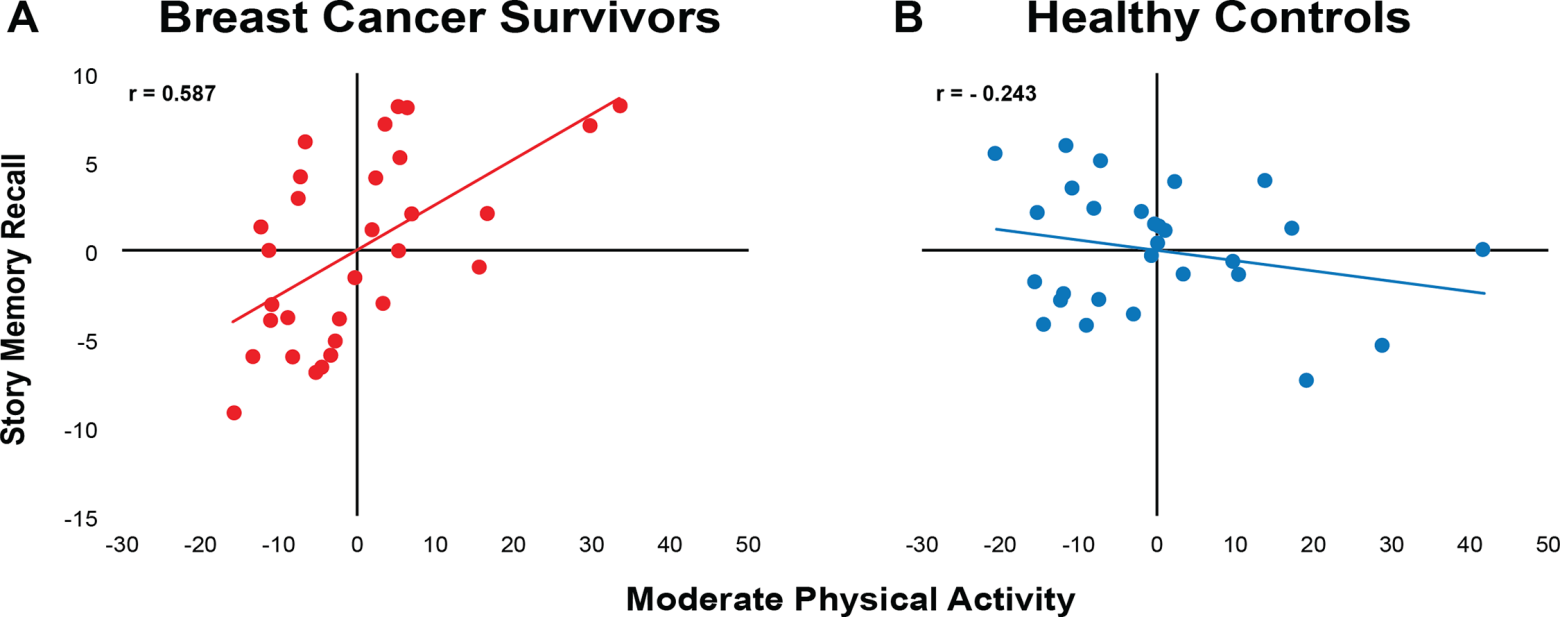
Scatterplots of unstandardized coefficients examining the relationship between story memory recall and moderate physical activity. Controlling for age and education, there was a positive relationship between story memory recall and moderate physical activity in the breast cancer survivors; greater story memory recall was significantly related to greater moderate physical activity (p<0.01), while there was no relationship in the non-cancer age-matched controls (p = 0.116).

In BCS, those who were more engaged in moderate PA had smaller WM lesion volumes (r = -0.430, p = 0.011); however, there was no relationship evident in the non-cancer age-matched controls (r = -0.120, p = 0.280; Fig 2). Furthermore, lower WM lesion volume in BCS was associated with greater story memory recall (r = -0.553, p = 0.001), while again there was also no relationship in the non-cancer age-matched controls (r = -0.075, p = 0.358; Fig 3). Lastly, BCS with greater levels of moderate PA performed better on the story memory recall (r = 0.587, p = 0.001), although this was not evident in non-cancer age-matched controls (r = -0.243, p = 0.116; Fig 4).

### Does moderate PA mediate the relationship between lesion volume and memory recall?

Given the relationships between lesion volume, moderate PA and story memory recall in BCS, and the lack of a relationship in non-cancer age-matched controls, the mediation analysis was conducted only in the BCS group. Our aim was to examine the potential mediation of moderate PA on the relationship between lesion volume and story memory recall.

Mediation analysis controlling for age and years of education indicated that lesion volume influenced story memory recall independent of its effect on moderate PA (c’ = -2.31). However, lesion volume also indirectly influenced story memory recall through its effect on moderate PA. BCS with lower lesion volume had higher levels of moderate PA (a = -6.14), and those with higher levels of moderate PA had greater recall of the story (b = 0.19). A bias-corrected bootstrap confidence interval for the indirect effect based on 10,000 bootstrap samples was entirely above zero (-0.0966 to -2.7069), verifying that moderate PA significantly mediated the impact of WM lesion volume on story memory recall in BCS (Fig 4).

### Does time since end of treatment impact the relationship between lesion volume, physical activity and memory recall in BCS?

While there were no differences between the treatment types in time since treatment completion for the BCS (Table 2), we wanted to ensure that the strong relationships between WM lesion volume, moderate PA, and story memory recall were not impacted by time since completion of treatment. Controlling for age, education and months since end of treatment, lesion volume was still negatively correlated with moderate PA (r = -0.455, p = 0.017) and story memory recall (r = -0.537, p = 0.004), and finally, story memory recall and moderate PA were positively related (r = 0.594, p = 0.001).

After re-running the mediation analysis controlling for age, years of education and months since end of treatment, we again confirmed that moderate PA significantly mediated the relationship between lesion volume and story memory recall in BCS. Lesion volume influenced story memory recall independent of its effect on moderate PA (c’ = -2.21). Lesion volume also indirectly influenced story memory recall through its effect on moderate PA. BCS with lower lesion volume had higher levels of moderate PA (a = -6.89), and those with higher levels of moderate PA had greater recall of the story (b = 0.19). A bias-corrected bootstrap confidence interval for the indirect effect based on 10,000 bootstrap samples was entirely above zero (-0.1821 to -3.2297). Despite the range in time since treatment (2–33 months), moderate PA significantly mediated the influence of WM lesion on story memory recall.

## Discussion

Previous studies have suggested that diagnosis and treatment for breast cancer results in cognitive impairments and structural changes in the brain, which may reflect a pattern of accelerated aging [3,9,12,38]. Given the association between PA and changes in brain structure and function in the aging literature [32–34,44,45], PA shows great potential for improving the long-term health of BCS, yet the link between PA, cognitive function and brain structure in BCS has received no attention. In this novel cross-sectional study, we examined the role that PA plays in the structure-function relationship in a group of middle-aged women within 3 years of completion of treatment, and non-cancer age-matched controls. Considering the small sample size, we compared across breast cancer as a whole rather then focusing on specific treatment types, investigating the link between memory recall, WM lesion volume and moderate PA. We controlled for both age and education throughout, as they are known to confound memory performance and structural integrity. Additional analyses also controlled for time since cessation of treatment in the BCS group only.

This study is the first to examine the link between WM lesions and PA in any cancer group. While there were no differences in WM lesion volume or moderate PA measures between the groups, we found that WM lesion volume was associated with moderate PA in BCS–those who were more active had fewer WM lesions. There was no relationship between these variables in the non-cancer age-matched controls. These results are particularly noteworthy given that we controlled for age, years of education, and in an additional analysis, time since end of treatment. Taken together, these suggest that moderate PA could play an important role in WM integrity following treatment for breast cancer. Previous work in older adults indicated that self-reported measures of PA predicted structural integrity, including WM lesion volume 3 years later [47]. They found that greater PA was related to lower WM lesion volume, higher WM integrity and higher GM volume. More importantly, using a similar objective PA measurement method as the present study, Burzynska and colleagues reported that greater moderate-vigorous PA was related to lower WM lesion volume in healthy but low-fit older adults [44]. In a group of normal older adults, Wirth and colleagues examined the interaction between cognition, brain pathology and PA [45], reporting a negative relationship between WM lesion volume and both cognition and self-reported PA. Some researchers have suggested that treatment for breast cancer may result in an accelerated pattern of aging in the brain [9], and PA interventions have been linked with improvements in brain structure and function in regions of the brain that are susceptible to aging, implying that breast cancer patients are likely to benefit greatly from a PA intervention.

Although the time since the end of treatment was relatively short in the current study, and the results may differ with a longer time span, they do indicate that treatment for breast cancer impacts the relationship between the structure and function of the brain. Several studies have investigated the link between changes in WM integrity and cognition following treatment for breast cancer [10,17,21,24,25,65], reporting a consistent pattern of degradation in WM integrity after treatment, which is often accompanied by cognitive impairments. Deprez and colleagues found a significant relationship between WM integrity and measures of attention and processing speed–where greater WM integrity was related to better attention and faster response time in BCS [21]. In a subsequent longitudinal follow-up, WM integrity was related to change in attention and verbal memory scores–greater decline in WM integrity following chemotherapy was related to greater decline in cognition [10]. While these studies did not explicitly measure WM lesion volume, they do support the findings from the current study that suggest greater WM integrity is related to greater cognitive function in BCS.

Cognitive function can be improved with increased PA across the lifespan [30,32,33]; however, there is little evidence of the link between PA and cognition using objective measures in BCS. We demonstrated that BCS who engaged in more moderate PA had better performance on story memory recall, using an objective measure of PA and cognition. Despite the range in time since treatment (2–33 months), this association was still significant when we controlled for time since end of treatment; this is particularly important as it suggests that PA has a positive influence throughout this period. Since BCS often decrease their level of exercise following diagnosis as well as during treatment, our findings may encourage BCS to maintain, or even increase their levels of PA during and following treatment.

While previous studies have found that gentle exercise and meditation improves self-reported cognitive function in cancer patients [66], and that rats that exercised post chemotherapy showed improved cognitive function in comparison to non-exercising rats [67], there are only 2 studies that have compared PA/fitness and cognition in BCS. Mackenzie and colleagues support differences in physical activity, heart rate recovery (an indicator of cardiorespiratory fitness), and working memory between breast cancer survivors and non-cancer age-matched controls. Greater cardiorespiratory fitness, heart rate recovery, and physical activity were positively associated with better working memory performance [56]. Furthermore, Crowgey and colleagues found that although fitness levels were lower in BCS, there was a positive relationship between fitness and visual memory [55]. Although the evidence of a significant link between PA and cognition in BCS is limited, previous studies, along with our findings, support an association between these variables, warranting further investigation in a large-scale longitudinal study.

One of the strengths of the current study is the use of objective measurements of PA, WM lesion volume and memory recall. However, the cross sectional nature of our study did not allow us to determine the impact of change in one or all of these variables; for example, would increasing PA by taking part in an exercise intervention, result in decreased WM lesions and increased memory recall. Yet, previous studies in healthy adults offer great promise–following a one-year walking intervention, Voss and colleagues reported that increased fitness in older adults who had completed an aerobic exercise program was significantly related to improved WM integrity [32]. This was largely apparent in regions of the brain that are known to show loss due to aging, and typically overlap with regions of the brain effected by treatment for breast cancer. Combined with our findings of a significant relationship between PA and WM lesions, this proposes a potentially modifiable lifestyle factor that could increase WM integrity in breast cancer patients undergoing treatment, as well as throughout their long-term survival.

While several studies have indicated that there are differences following diagnosis of breast cancer but before treatment [68,69], our design did not allow us to examine these. Additionally, given the novelty of our study, we did not compare across treatment types. Recently, studies assessing the effects of breast cancer treatment type compared high-dose (HCh+) and conventional-dose chemotherapy (CC+), radiation (RT) only and non-cancer age-matched controls [70,71]. They reported cognitive decline in the HCh+ group, with diminished impairment evident in the CC+ and RT groups; gray matter (GM) volume reductions for both the HCh+ and CC+ groups when compared to RT only; reductions in WM integrity only in the HCh+ group; and hypoactivation in task-related performance in both chemotherapy groups, though this was more pronounced in the HCh+ group. These findings suggest that there are treatment dependent differences in GM volume, WM integrity and cognitive function following therapy for breast cancer that deserves further investigation.

### Conclusion

There are modifiable risk factors associated with treatment related changes in brain structure and function, and while there is evidence indicating change in cognitive function, WM integrity and PA in BCS, there are no studies that have directly compared all three variables in this group. Our novel findings suggest that continued physical activity might be particularly important in BCS as brain structure significantly predicted cognitive function via mediation of physical activity. Continually active lifestyles may help reduce the treatment related vulnerabilities associated with breast cancer, including reduced WM integrity and cognitive impairment. Collectively, these findings suggest that PA plays an important role in both brain structure and function, warranting further investigation in larger PA intervention studies.

## Supporting Information

S1 Dataset Dataset from all participants. Demographic information, moderate physical activity, lesion volume, and story memory recall data for each subject included in the analysis (n = 58). Data set also includes disease and treatment characteristics of breast cancer survivors (n = 30).
(XLSX)

## References

[1] Siegel RL, Miller KD, Jemal A (2015) Cancer statistics, 2015. CA Cancer J Clin 65: 5–29. 10.3322/caac.21254 25559415

[2] Society AC (2015) "What are the key statistics about breast cancer?".

[3] Nguyen CM, Yamada TH, Beglinger LJ, Cavanaugh JE, Denburg NL, Schultz SK (2013) Cognitive Features Ten or More Years After Successful Breast Cancer Survival: Comparisons Across Types of Cancer Interventions. Psychooncology 22: 862–868. 10.1002/pon.3086 22585465 PMC3475736

[4] Siegel R, Ma J, Zou Z, Jemal A (2014) Cancer statistics, 2014. CA Cancer J Clin 64: 9–29. 10.3322/caac.21208 24399786

[5] Siegel R, Naishadham D, Jemal A (2013) Cancer statistics, 2013. CA Cancer J Clin 63: 11–30. 10.3322/caac.21166 23335087

[6] Ahles TA, Root JC, Ryan EL (2012) Cancer- and cancer treatment-associated cognitive change: an update on the state of the science. J Clin Oncol 30: 3675–3686. 10.1200/JCO.2012.43.0116 23008308 PMC3675678

[7] Ahles TA, Saykin AJ, McDonald BC, Furstenberg CT, Cole BF, Hanscom BS, et al (2008) Cognitive function in breast cancer patients prior to adjuvant treatment. Breast Cancer Res Treat 110: 143–152. 17674194 10.1007/s10549-007-9686-5 PMC3114441

[8] Wefel JS, Lenzi R, Theriault R, Buzdar AU, Cruickshank S, Meyers CA (2004) 'Chemobrain' in breast carcinoma?: a prologue. Cancer 101: 466–475. 15274059 10.1002/cncr.20393

[9] Mandelblatt JS, Hurria A, McDonald BC, Saykin AJ, Stern RA, VanMeter JW, et al (2013) Cognitive effects of cancer and its treatments at the intersection of aging: what do we know; what do we need to know? Semin Oncol 40: 709–725. 10.1053/j.seminoncol.2013.09.006 24331192 PMC3880205

[10] Deprez S, Amant F, Smeets A, Peeters R, Leemans A, Van Hecke W, et al (2012) Longitudinal assessment of chemotherapy-induced structural changes in cerebral white matter and its correlation with impaired cognitive functioning. J Clin Oncol 30: 274–281. 10.1200/JCO.2011.36.8571 22184379

[11] Yamada TH, Denburg NL, Beglinger LJ, Schultz SK (2010) Neuropsychological outcomes of older breast cancer survivors: cognitive features ten or more years after chemotherapy. J Neuropsychiatry Clin Neurosci 22: 48–54. 10.1176/appi.neuropsych.22.1.48 20160209 PMC3641161

[12] Wefel JS, Kesler SR, Noll KR, Schagen SB (2015) Clinical characteristics, pathophysiology, and management of noncentral nervous system cancer-related cognitive impairment in adults. CA Cancer J Clin 65: 123–138. 10.3322/caac.21258 25483452 PMC4355212

[13] Kaiser J, Bledowski C, Dietrich J (2014) Neural correlates of chemotherapy-related cognitive impairment. Cortex 54: 33–50. 10.1016/j.cortex.2014.01.010 24632463

[14] Simo M, Rifa-Ros X, Rodriguez-Fornells A, Bruna J (2013) Chemobrain: a systematic review of structural and functional neuroimaging studies. Neurosci Biobehav Rev 37: 1311–1321. 10.1016/j.neubiorev.2013.04.015 23660455

[15] Saykin AJ, de Ruiter MB, McDonald BC, Deprez S, Silverman DH (2013) Neuroimaging biomarkers and cognitive function in non-CNS cancer and its treatment: current status and recommendations for future research. Brain Imaging Behav 7: 363–373. 10.1007/s11682-013-9283-7 24327327 PMC3909524

[16] Yoshikawa E, Matsuoka Y, Inagaki M, Nakano T, Akechi T, Kobayakawa M, et al (2005) No adverse effects of adjuvant chemotherapy on hippocampal volume in Japanese breast cancer survivors. Breast Cancer Res Treat 92: 81–84. 15980995 10.1007/s10549-005-1412-6

[17] Koppelmans V, de Groot M, de Ruiter MB, Boogerd W, Seynaeve C, Vernooij MW, et al (2014) Global and focal white matter integrity in breast cancer survivors 20 years after adjuvant chemotherapy. Hum Brain Mapp 35: 889–899. 10.1002/hbm.22221 23281152 PMC6869525

[18] Koppelmans V, Vernooij MW, Boogerd W, Seynaeve C, Ikram MA, Breteler MM, et al (2015) Prevalence of cerebral small-vessel disease in long-term breast cancer survivors exposed to both adjuvant radiotherapy and chemotherapy. J Clin Oncol 33: 588–593. 10.1200/JCO.2014.56.8345 25559803

[19] Kesler S, Janelsins M, Koovakkattu D, Palesh O, Mustian K, Morrow G, et al (2013) Reduced hippocampal volume and verbal memory performance associated with interleukin-6 and tumor necrosis factor-alpha levels in chemotherapy-treated breast cancer survivors. Brain Behav Immun 30 Suppl: S109–116. 10.1016/j.bbi.2012.05.017 22698992 PMC3665606

[20] McDonald BC, Saykin AJ (2013) Alterations in brain structure related to breast cancer and its treatment: chemotherapy and other considerations. Brain Imaging Behav 7: 374–387. 10.1007/s11682-013-9256-x 23996156 PMC3869865

[21] Deprez S, Amant F, Yigit R, Porke K, Verhoeven J, Van den Stock J, et al (2011) Chemotherapy-induced structural changes in cerebral white matter and its correlation with impaired cognitive functioning in breast cancer patients. Hum Brain Mapp 32: 480–493. 10.1002/hbm.21033 20725909 PMC6870393

[22] Fields RD (2010) Change in the Brain’s White Matter: The role of the brain’s white matter in active learning and memory may be underestimated. Science (New York, NY) 330: 768–769. 10.1126/science.1199139 PMC3201847 21051624

[23] Deprez S, Billiet T Fau—Sunaert S, Sunaert S Fau—Leemans A, Leemans A Diffusion tensor MRI of chemotherapy-induced cognitive impairment in non-CNS cancer patients: a review. 10.1007/s11682-012-9220-1 23329357

[24] de Ruiter MB, Reneman L, Boogerd W, Veltman DJ, Caan M, Douaud G, et al (2012) Late effects of high-dose adjuvant chemotherapy on white and gray matter in breast cancer survivors: converging results from multimodal magnetic resonance imaging. Hum Brain Mapp 33: 2971–2983. 10.1002/hbm.21422 22095746 PMC6870296

[25] Koppelmans V, de Ruiter MB, van der Lijn F, Boogerd W, Seynaeve C, van der Lugt A, et al (2012) Global and focal brain volume in long-term breast cancer survivors exposed to adjuvant chemotherapy. Breast Cancer Res Treat 132: 1099–1106. 10.1007/s10549-011-1888-1 22205140

[26] Erickson KI, Prakash RS, Voss MW, Chaddock L, Heo S, McLaren M, et al (2010) Brain-derived neurotrophic factor is associated with age-related decline in hippocampal volume. J Neurosci 30: 5368–5375. 10.1523/JNEUROSCI.6251-09.2010 20392958 PMC3069644

[27] Erickson KI, Prakash RS, Voss MW, Chaddock L, Hu L, Morris KS, et al (2009) Aerobic fitness is associated with hippocampal volume in elderly humans. Hippocampus 19: 1030–1039. 10.1002/hipo.20547 19123237 PMC3072565

[28] Erickson KI, Raji CA, Lopez OL, Becker JT, Rosano C, Newman AB, et al (2010) Physical activity predicts gray matter volume in late adulthood: the Cardiovascular Health Study. Neurology 75: 1415–1422. 10.1212/WNL.0b013e3181f88359 20944075 PMC3039208

[29] Colcombe SJ, Erickson KI, Raz N, Webb AG, Cohen NJ, McAuley E, et al (2003) Aerobic fitness reduces brain tissue loss in aging humans. J Gerontol A Biol Sci Med Sci 58: 176–180. 12586857 10.1093/gerona/58.2.m176

[30] Colcombe SJ, Kramer AF, McAuley E, Erickson KI, Scalf P (2004) Neurocognitive aging and cardiovascular fitness: recent findings and future directions. J Mol Neurosci 24: 9–14. 15314244 10.1385/JMN:24:1:009

[31] Voss MW, Erickson KI, Prakash RS, Chaddock L, Malkowski E, Alves H, et al (2010) Functional connectivity: a source of variance in the association between cardiorespiratory fitness and cognition? Neuropsychologia 48: 1394–1406. 10.1016/j.neuropsychologia.2010.01.005 20079755 PMC3708614

[32] Voss MW, Heo S, Prakash RS, Erickson KI, Alves H, Chaddock L, et al (2013) The influence of aerobic fitness on cerebral white matter integrity and cognitive function in older adults: results of a one-year exercise intervention. Hum Brain Mapp 34: 2972–2985. 10.1002/hbm.22119 22674729 PMC4096122

[33] Erickson KI, Voss MW, Prakash RS, Basak C, Szabo A, Chaddock L, et al (2011) Exercise training increases size of hippocampus and improves memory. Proc Natl Acad Sci U S A 108: 3017–3022. 10.1073/pnas.1015950108 21282661 PMC3041121

[34] Kramer AF, Erickson KI (2007) Capitalizing on cortical plasticity: influence of physical activity on cognition and brain function. Trends Cogn Sci 11: 342–348. 17629545 10.1016/j.tics.2007.06.009

[35] Mandelblatt JS, Stern RA, Luta G, McGuckin M, Clapp JD, Hurria A, et al (2014) Cognitive impairment in older patients with breast cancer before systemic therapy: is there an interaction between cancer and comorbidity? J Clin Oncol 32: 1909–1918. 10.1200/JCO.2013.54.2050 24841981 PMC4050204

[36] Sanoff HK, Deal AM, Krishnamurthy J, Torrice C, Dillon P, Sorrentino J, et al (2014) Effect of cytotoxic chemotherapy on markers of molecular age in patients with breast cancer. J Natl Cancer Inst 106: dju057 10.1093/jnci/dju057 24681605 PMC3982894

[37] Henderson TO, Ness KK, Cohen HJ (2014) Accelerated aging among cancer survivors: from pediatrics to geriatrics. Am Soc Clin Oncol Educ Book: e423–430. 10.14694/EdBook_AM.2014.34.e423 24857133

[38] Schmitz KH, Cappola AR, Stricker CT, Sweeney C, Norman SA (2007) The intersection of cancer and aging: establishing the need for breast cancer rehabilitation. Cancer Epidemiol Biomarkers Prev 16: 866–872. 17507607 10.1158/1055-9965.EPI-06-0980

[39] Kempermann G, Fabel K, Ehninger D, Babu H, Leal-Galicia P, Garthe A, et al (2010) Why and how physical activity promotes experience-induced brain plasticity. Front Neurosci 4: 189 10.3389/fnins.2010.00189 21151782 PMC3000002

[40] Courneya KS, Segal RJ, Gelmon K, Reid RD, Mackey JR, Friedenreich CM, et al (2007) Six-month follow-up of patient-rated outcomes in a randomized controlled trial of exercise training during breast cancer chemotherapy. Cancer Epidemiol Biomarkers Prev 16: 2572–2578. 18086760 10.1158/1055-9965.EPI-07-0413

[41] Courneya KS, Segal RJ, Mackey JR, Gelmon K, Reid RD, Friedenreich CM, et al (2007) Effects of aerobic and resistance exercise in breast cancer patients receiving adjuvant chemotherapy: a multicenter randomized controlled trial. J Clin Oncol 25: 4396–4404. 17785708 10.1200/JCO.2006.08.2024

[42] Galantino ML, Greene L, Daniels L, Dooley B, Muscatello L, O'Donnell L (2012) Longitudinal impact of yoga on chemotherapy-related cognitive impairment and quality of life in women with early stage breast cancer: a case series. Explore (NY) 8: 127–135. 22385567 10.1016/j.explore.2011.12.001

[43] Mustian KM, Sprod LK, Janelsins M, Peppone LJ, Mohile S (2012) Exercise Recommendations for Cancer-Related Fatigue, Cognitive Impairment, Sleep problems, Depression, Pain, Anxiety, and Physical Dysfunction: A Review. Oncol Hematol Rev 8: 81–88. 23667857 10.17925/ohr.2012.08.2.81 PMC3647480

[44] Burzynska AZ, Chaddock-Heyman L, Voss MW, Wong CN, Gothe NP, Olson EA, et al (2014) Physical activity and cardiorespiratory fitness are beneficial for white matter in low-fit older adults. PLoS One 9: e107413 10.1371/journal.pone.0107413 25229455 PMC4167864

[45] Wirth M, Haase CM, Villeneuve S, Vogel J, Jagust WJ (2014) Neuroprotective pathways: lifestyle activity, brain pathology, and cognition in cognitively normal older adults. Neurobiol Aging 35: 1873–1882. 10.1016/j.neurobiolaging.2014.02.015 24656834 PMC4019766

[46] Spence RR, Heesch KC, Brown WJ (2010) Exercise and cancer rehabilitation: a systematic review. Cancer Treat Rev 36: 185–194. 10.1016/j.ctrv.2009.11.003 19962830

[47] Gow AJ, Bastin ME, Munoz Maniega S, Valdes Hernandez MC, Morris Z, Murray C, et al (2012) Neuroprotective lifestyles and the aging brain: activity, atrophy, and white matter integrity. Neurology 79: 1802–1808. 10.1212/WNL.0b013e3182703fd2 23091073

[48] Irwin ML, Crumley D, McTiernan A, Bernstein L, Baumgartner R, Gilliland FD, et al (2003) Physical activity levels before and after a diagnosis of breast carcinoma: the Health, Eating, Activity, and Lifestyle (HEAL) study. Cancer 97: 1746–1757. 12655532 10.1002/cncr.11227 PMC3034406

[49] Jones LW, Eves ND, Haykowsky M, Freedland SJ, Mackey JR (2009) Exercise intolerance in cancer and the role of exercise therapy to reverse dysfunction. Lancet Oncol 10: 598–605. 10.1016/S1470-2045(09)70031-2 19482248

[50] Sabiston CM, Brunet J, Vallance JK, Meterissian S (2014) Prospective examination of objectively assessed physical activity and sedentary time after breast cancer treatment: sitting on the crest of the teachable moment. Cancer Epidemiol Biomarkers Prev 23: 1324–1330. 10.1158/1055-9965.EPI-13-1179 24753546

[51] McGuire DK, Levine BD, Williamson JW, Snell PG, Blomqvist CG, Saltin B, et al (2001) A 30-year follow-up of the Dallas Bedrest and Training Study: II. Effect of age on cardiovascular adaptation to exercise training. Circulation 104: 1358–1366. 11560850

[52] McGuire DK, Levine BD, Williamson JW, Snell PG, Blomqvist CG, Saltin B, et al (2001) A 30-year follow-up of the Dallas Bedrest and Training Study: I. Effect of age on the cardiovascular response to exercise. Circulation 104: 1350–1357. 11560849

[53] Schmitz KH, Courneya KS, Matthews C, Demark-Wahnefried W, Galvao DA, Pinto BM, et al (2010) American College of Sports Medicine roundtable on exercise guidelines for cancer survivors. Med Sci Sports Exerc 42: 1409–1426. 10.1249/MSS.0b013e3181e0c112 20559064

[54] Chandwani KD, Perkins G, Nagendra HR, Raghuram NV, Spelman A, Nagarathna R, et al (2014) Randomized, controlled trial of yoga in women with breast cancer undergoing radiotherapy. J Clin Oncol 32: 1058–1065. 10.1200/JCO.2012.48.2752 24590636 PMC3965260

[55] Crowgey T, Peters KB, Hornsby WE, Lane A, McSherry F, Herndon JE 2nd, et al (2014) Relationship between exercise behavior, cardiorespiratory fitness, and cognitive function in early breast cancer patients treated with doxorubicin-containing chemotherapy: a pilot study. Appl Physiol Nutr Metab 39: 724–729. 10.1139/apnm-2013-0380 24869976 PMC4542053

[56] Mackenzie MJ, Zuniga K. E., Raine L. B., Awick E. A., Hillman C. H., Kramer A., F. M, E. (Under review) Cardiorespiratory fitness, heart rate recovery, physical activity, and working memory in breast cancer survivors and agematched controls. Journal of Women’s Health. 10.1089/jwh.2015.5246 PMC4741207 26418463

[57] Folstein MF, Folstein, S.E., White, T., Messer, M.A. (2010) Mini-Mental State Examination, 2nd Edition: Users Manual. Lutz, FL: Psychological Assessment Resources.

[58] Smith SM, Jenkinson M, Woolrich MW, Beckmann CF, Behrens TEJ, Johansen-Berg H, et al (2004) Advances in functional and structural MR image analysis and implementation as FSL. Neuroimage 23: S208–S219. 15501092 10.1016/j.neuroimage.2004.07.051

[59] Wetter NC, Hubbard, E.A., Motl, R.W., Sutton B.P. (2015) T2 WM Hyperintensity Mapping and Quantification With FSL. Intl Society for Magnetic Resonance in Medicine. Toronto, Canada. pp. 1408.

[60] Smith SM (2002) Fast robust automated brain extraction. Human Brain Mapping 17: 143–155. 12391568 10.1002/hbm.10062 PMC6871816

[61] Zhang YY, Brady M, Smith S (2001) Segmentation of brain MR images through a hidden Markov random field model and the expectation-maximization algorithm. Ieee Transactions on Medical Imaging 20: 45–57. 11293691 10.1109/42.906424

[62] Andersson JL, Jenkinson M, Smith S (2007) Non-linear registration, aka Spatial normalisation FMRIB technical report TR07JA2 FMRIB Analysis Group of the University of Oxford.

[63] Hayes AF (2013) Introduction to Mediation, Moderation, and Conditional Process Analysis: A Regression-Based Approach New York, NY: The Guilford Press.

[64] Rucker DD, Preacher KJ, Tormala ZL, Petty RE (2011) Mediation Analysis in Social Psychology: Current Practices and New Recommendations. Social and Personality Psychology Compass 5: 359–371.

[65] Abraham J, Haut MW, Moran MT, Filburn S, Lemiuex S, Kuwabara H (2008) Adjuvant chemotherapy for breast cancer: effects on cerebral white matter seen in diffusion tensor imaging. Clin Breast Cancer 8: 88–91. 10.3816/CBC.2008.n.007 18501063

[66] Oh B, Butow PN, Mullan BA, Clarke SJ, Beale PJ, Pavlakis N, et al (2012) Effect of medical Qigong on cognitive function, quality of life, and a biomarker of inflammation in cancer patients: a randomized controlled trial. Support Care Cancer 20: 1235–1242. 10.1007/s00520-011-1209-6 21688163

[67] Fardell JE, Vardy J, Shah JD, Johnston IN (2012) Cognitive impairments caused by oxaliplatin and 5-fluorouracil chemotherapy are ameliorated by physical activity. Psychopharmacology (Berl) 220: 183–193. 21894483 10.1007/s00213-011-2466-2

[68] Scherling C, Collins B, Mackenzie J, Bielajew C, Smith A (2012) Prechemotherapy differences in response inhibition in breast cancer patients compared to controls: a functional magnetic resonance imaging study. J Clin Exp Neuropsychol 34: 543–560. 10.1080/13803395.2012.666227 22380580

[69] Scherling C, Collins B, MacKenzie J, Bielajew C, Smith A (2011) Pre-chemotherapy differences in visuospatial working memory in breast cancer patients compared to controls: An fMRI study. Frontiers in Human Neuroscience 5. 10.3389/fnhum.2011.00122 PMC3205481 22053153

[70] Stouten-Kemperman MM, de Ruiter MB, Koppelmans V, Boogerd W, Reneman L, Schagen SB (2014) Neurotoxicity in breast cancer survivors >/ = 10 years post-treatment is dependent on treatment type. Brain Imaging Behav. 10.1007/s11682-014-9305-0 24858488

[71] Stouten-Kemperman MM, de Ruiter MB, Boogerd W, Veltman DJ, Reneman L, Schagen SB (2014) Very Late Treatment-Related Alterations in Brain Function of Breast Cancer Survivors. J Int Neuropsychol Soc: 1–12. 10.1017/S1355617714001015 25529014

